# The hidden connection: systemic immune-inflammation index and its role in asthma

**DOI:** 10.1016/j.clinsp.2025.100779

**Published:** 2025-09-06

**Authors:** Cheng Peng, Dong Gao, Zanchen Zhou, Tiancheng Wang

**Affiliations:** a School of Pharmacy, Anhui University of Chinese Medicine, China; b School of Nursing, Anhui University of Chinese Medicine, China; c The First Clinical School of Anhui University of Chinese Medicine, China; d School of Integrated Traditional Chinese and Western Medicine, Anhui University of Chinese Medicine, China

**Keywords:** Systemic Immune-Inflammation Index, Asthma, Cross-sectional study, Population-based research, NHANES

## Abstract

**Background:**

This study explores potential associations between asthma and the Systemic Immune-Inflammation Index (SII).

**Methods:**

The study primarily focuses on adults with complete data on SII and asthma from the National Health and Nutrition Examination Survey database (2015–2020). SII is calculated using the formula: platelet count × neutrophil count / lymphocyte count. The connection between SII and asthma was assessed through baseline feature analysis, weighted multivariate regression, subgroup analysis, and smooth curve fitting.

**Results:**

A total of 13,334 participants were included, with 2045 individuals (15.34 %) having asthma. Subgroup analysis and interaction tests revealed no significant impact of gender, age, body mass index, vitamin C, magnesium, coronary heart disease, or diabetes on the observed association (p for interaction > 0.05).

**Conclusion:**

Higher SII levels are associated with an increased incidence of asthma. Further prospective studies are needed to confirm these findings.

## Highlights

• The increase in SII level is associated with an increase in asthma incidence.
• Age disparities may influence the relationship between inflammation and asthma.
• Higher SII correlates with a 31 % increased asthma incidence.

## Introduction

Asthma is a chronic respiratory disease characterized by airway inflammation, mucus overproduction, and bronchospasm, leading to breathing difficulties.^1^ It affects over 35 billion people globally,^2^ with higher incidence and comorbidities observed among older adults in the US.^3^^,^^4^ According to the Global Asthma Initiative and national guidelines, asthma is categorized by severity to guide treatment strategies.^5^ In the UK, it is prevalent among adolescents, with exercise-based, non-pharmacologic interventions proving beneficial. The Systemic Immune-Inflammation Index (SII), calculated as platelet × neutrophil / lymphocyte count, reflects systemic and local immune status.^6^^,^^7^ Given emerging evidence of immune involvement in asthma,^8^ a potential association between SII and asthma warrants investigation.

Kristie R. Ross et al. conducted a three-year study involving 111 children and reported that those with peripheral eosinophil counts >436 cells/µL had a 2.75-fold increased odds of asthma resolution.^9^ M Canöz et al. explored the relationship between inflammatory markers, asthma, and obesity, revealing significantly elevated levels of leptin, C-Reactive Protein (CRP), erythrocyte sedimentation rate, Tumor Necrosis Factor-α (TNF-α), and Interleukin-6 (IL-6) in patients compared to healthy controls (*p* < 0.01).^10^ Bronwyn S. Berthon et al. examined the impact of fruit and vegetable intake on childhood asthma, assessing TNF-α, CRP, IL-6, and peripheral blood mononuclear cells as secondary outcomes, but found that a high-fruit and vegetable diet did not significantly affect asthma exacerbations.^11^ These studies highlight the frequent inclusion of inflammatory markers as outcome variables in asthma research. However, the Systemic Immune-Inflammation Index (SII), a novel and integrated marker of inflammation, has not yet been studied in relation to asthma, and its potential association remains unclear.

To explore the association between SII and asthma, this study aims to analyze data from the National Health and Nutrition Examination Survey (NHANES) in the United States. The authors hypothesize that elevated SII levels are associated with an increased prevalence of asthma.

## Materials and methods

### Data sources and participants

**Fig. 1:**
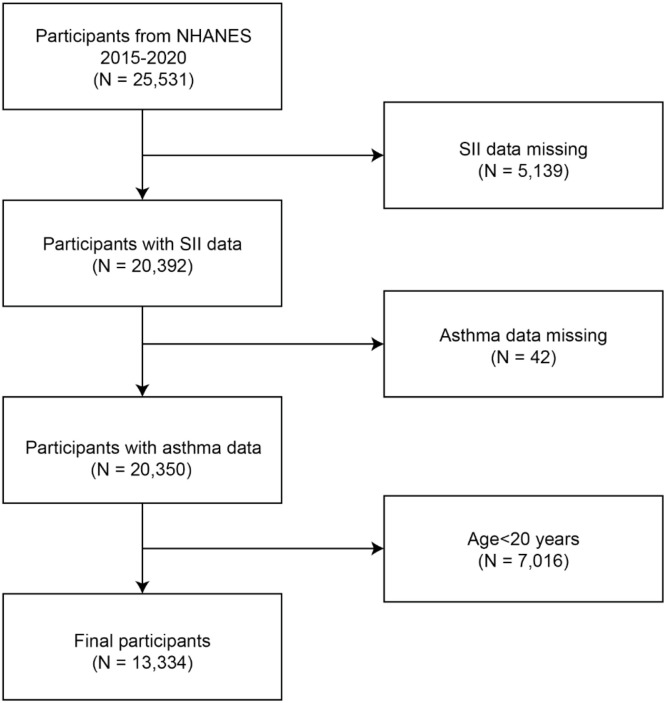
Flowchart of the study participant selection.

The NHANES database provides information, which assesses the health and eating habits of the American populace with an emphasis on various population subgroups or health themes.^12^ This study has the advantage of incorporating demographic, diet, examination, laboratory examination, and questionnaire data in addition to interviews and physical tests. Medical personnel do the checkup. You can get further information and an introduction on the website https://www.cdc.gov/nchs/nhanes/, which is widely accessible. To investigate the connection between SII and asthma, the authors chose three database cycles between 2015 and 2018. The exclusion criteria for the study are: 1) Age < 20 years old, 2) Lack of asthma questionnaire data, and 3) Lack of SII data. Firstly, 25,531 people were included in the 2015‒2020 data, 5199 people were missing from the SII data, 42 people were missing from the asthma data, and 7016 people were under the age of 20, as shown in Fig. 1. Finally, a total of 13,334 people were included in the complete study data.

### Exposure and outcome variables

The exposure variable is SII, and the DxH 800 Coulter analyzer is used to measure lymphocyte, neutrophil, and plate counts through whole blood cell counting. The calculation formula is Platelet count × Neutrophil count / Lymphocyte count, in units of 1000 cells/µL.^7^ The NHANES database questionnaire data, medical problems, and whether or not doctors or other healthcare providers have informed you that you have asthma are all used to calculate the outcome variable.

### Covariates

Based on previous studies, the authors included the following variables in this experiment according to study SII and asthma.^13^^,^^14^ The following variables were included: age, gender, race, marital status, fish ate during the past 30 days, high blood pressure, diabetes, cancer or malignancy, smoked, ratio of family income to poverty, protein, dietary fiber, cholesterol, vitamin C, magnesium, zinc, weight, Body Mass Index (BMI), total cholesterol, fasting glucose, cholesterol, triglycerides, uric acid, alcohol drink. For the identification of whether to smoke, the authors choose indicators: accumulating 100 cigarettes in a lifetime is considered a smoker, and <100 cigarettes is considered a non-smoker.^15^ The collection criteria for these covariates are recorded on the official website of the database: https://www.cdc.gov/nchs/nhanes/.

### Statistical analysis

Under the direction of the Centers for Disease Control and Prevention, statistical analysis was carried out utilizing sampling weights from the NHANES database, and sophisticated multi-stage clustering surveys were also taken into consideration. Categorical data are displayed in proportion, while continuous variables are displayed in standard deviation. The Chi-Square test and variance-weighted analysis were used to examine group differences. Test the relationship between SII and asthma using multivariate logistic regression in various models. Model 2 adjusted for age, gender, and race, while Model 3 adjusted for age, gender, race, Marital status, Fish eaten during the past 30 days, high blood pressure, diabetes, cancer or malignancy, smoked, ratio of family income to poverty, protein, dietary fiber, cholesterol, vitamin C, magnesium, zinc, weight, bmi, total cholesterol, fasting glucose, cholesterol, triglycerides, uric acid, alcohol drink make adjustments. To enhance interpretability and ensure numerical stability in regression analyses, the authors divided SII by 100. Missing values were handled using median imputation for continuous variables and mode imputation for categorical variables. Given that the proportion of missing data was low across all variables, this approach is considered appropriate and has been commonly applied in analyses of NHANES data. References to prior studies using similar imputation strategies have been added for justification. Subgroup analyses of the association between SII and asthma were performed stratified by age, sex, race, hypertension, diabetes, cancer and tumors, alcohol consumption, total energy, total sugar, vitamin C, magnesium, BMI, fasting glucose, and sugar hemoglobin. Apply three models, and construct multivariate tests by controlling variables and fitting smooth curves. Missing values are filled according to the median of existing continuous variables or the mode of logistic variables. All analyses using R4.1.0 and Empower2.0. *p* < 0.05 indicates significant data.

## Results

### Baseline characteristic

This study included a total of 13,334 adults based on the principles of inclusion and exclusion criteria, with an average age of 50.51±17.56 years in participants. Among the study population, 6428 males accounted for 48.21 %, and 6906 females accounted for 51.79 %; A total of 1886 Mexican Americans accounted for 14.14 %, 4563 non-Hispanic white people accounted for 34.22 %, 3174 non-Hispanic black people accounted for 23.80 %, and 3171 other races accounted for 27.83 %. Among them, 2045 people suffer from asthma (15.34 %), and 11,289 people do not have asthma. The average concentration of SII is 519.01±345.33 (1000 cells/µL).

Table 1 uses asthma as a column-stratified variable, and all covariates included in the study have statistical significance (*p* < 0.05). Compared with non-asthma patients, asthma patients tend to be younger, female, unmarried, with a lower ratio of family income to power, lower protein (gm) intake, lower cholesterol (mg) level, higher weight (kg), higher BMI (kg/m^2^), higher triglycerides (mg/dL) level, and higher SII level.

**Table 1:** Weighted features of Asthma-based research population.

Characteristic	Non-asthma formers (*n* = 11,289)	Asthma formers (*n* = 2045)	p-value
Age (year)	50.87 ± 17.52	48.55 ± 17.61	<0.001
Gender ( %)			<0.001
Male	49.36	41.86	
Female	50.64	58.14	
Race / Ethnicity ( %)			<0.001
Mexican American	15.05	9.14	
Non-Hispanic White	33.99	35.50	
Non-Hispanic Black	23.05	27.97	
Other	28.91	27.39	
Marital status ( %)			<0.001
Married	56.10	50.56	
Other	43.30	49.44	
Unclear	0.60	0.00	
Fish eaten during past 30-days ( %)			0.011
Yes	68.73	65.84	
No	29.83	33.21	
Unclear	1.44	0.95	
High blood pressure ( %)			<0.001
Yes	36.92	43.18	
No	62.96	56.72	
Unclear	0.12	0.10	
Diabetes ( %)			0.004
Yes	14.93	18.00	
No	82.35	79.12	
Unclear	2.72	2.88	
Cancer or malignancy ( %)			<0.001
Yes	9.81	11.83	
No	90.16	87.97	
Unclear	0.04	0.20	
Smoked ( %)			<0.001
Yes	40.92	47.09	
No	59.08	52.91	
Ratio of family income to poverty	2.56 ± 1.61	2.38 ± 1.62	<0.001
Protein (gm)	80.32 ± 42.28	77.16 ± 43.64	<0.001
Dietary fiber (gm)	16.94 ± 10.83	15.27 ± 9.82	<0.001
Cholesterol (mg)	314.83 ± 255.76	301.15 ± 251.24	0.007
Vitamin C (mg)	79.65 ± 89.60	77.10 ± 110.05	0.001
Magnesium (mg)	296.31 ± 151.60	281.64 ± 147.66	<0.001
Zinc (mg)	10.70 ± 8.35	10.15 ± 6.44	<0.001
Weight (kg)	82.20 ± 21.81	86.90 ± 25.53	<0.001
Body Mass Index (kg/m^2^)	29.56 ± 6.98	31.44 ± 8.87	<0.001
Total Cholesterol (mg/dL)	187.86 ± 41.04	185.43 ± 42.87	0.002
Fasting Glucose (mg/dL)	113.10 ± 37.95	116.29 ± 46.36	0.018
Cholesterol (mg/dL)	189.22 ± 41.65	186.70 ± 43.50	0.002
Triglycerides (mg/dL)	147.52 ± 116.99	141.14 ± 123.15	0.002
Uric acid (mg/dL)	5.41 ± 1.47	5.37 ± 1.44	0.231
Alcohol drink	4.50 ± 19.55	4.24 ± 4.11	0.029
SII	513.00 ± 325.08	552.25 ± 439.41	<0.001

The mean ± SD of continuous variables: *p*-value is calculated using a weighted linear regression model. % for Categorical variable: p-value is calculated by the weighted chi-square test.

Table A1 uses the quartile of SII as the column-stratified variable, and the covariates in the table have statistical significance (*p* < 0.05) except for whether or not they drink alcohol. Participants who fell into the Quarter 4 group ended up Female, non-Hispanic White, Fish eaters during the past 30 days, high weight, high BMI, and suffering from asthma. Compared with participants in Quartile 1, participants in Quartile 4 is older, have more women, have more hypertension, diabetes, and cancer, and have more smokers.

### Association between SII and Asthma

Table 2 shows the multivariate regression analysis of Asthma and SII/100. The research uses SII/100, which makes the SII effect size increase 100 times, and solves the problem that the effect size is not obvious. Among the three models, SII and Asthma are significantly correlated. Each 100-unit increase in the SII was associated with a 31 % increase in the incidence of asthma.

**Table 2:** The multivariate regression analysis of Asthma and SII/100.

	Crude Model (Model 1)	Partially Adjusted Model (Model 2)	Fully Adjusted Model (Model 3)
	OR (95 % CI) p-value	OR (95 % CI) p-value	OR (95 % CI) p-value
SII/100	1.03 (1.02, 1.04)^a^	1.03 (1.02, 1.04)^a^	1.03 (1.01, 1.04)^a^
SII/100 quartiles			
Quartile 1	Reference	Reference	Reference
Quartile 2	1.08 (0.94, 1.24)	1.11 (0.96, 1.28)	1.16 (0.99, 1.35)
Quartile 3	1.15 (1.00, 1.32)^b^	1.18 (1.02, 1.36)^b^	1.20 (1.02, 1.40)^b^
Quartile 4	1.29 (1.13, 1.48)^a^	1.31 (1.14, 1.50)^a^	1.31 (1.13, 1.53)^a^
p for trend	0.0001	0.0001	0.0010

Model 1, no covariates were adjusted. Model 2, age, sex, and race were adjusted. Model 3, age, gender, race, Marital status, Fish eaten during the past 30-days, High blood pressure, Diabetes, Cancer or malignancy, Smoked, Ratio of family income to poverty, Protein, Dietary fiber, Cholesterol, Vitamin C, Magnesium, Zinc, Weight, BMI, Total Cholesterol, Fasting Glucose, Cholesterol, Triglycerides, Uric acid, Alcohol drink were adjusted. 95 % CI, 95 % Confidence Interval; OR, Odds Ratio; SII, Systemic Immunity-Inflammation Index.

a p < 0.001.

b p < 0.05, ** *p* < 0.01,; *p* < 0.05 was considered statistically significant.

The total energy, total sugar, vitamin C, magnesium, triglyceride, total cholesterol, fasting blood sugar, and other laboratory indicators of respondents on the first day were not obvious, so the authors chose to divide the above indicators by 100 to amplify the size of the effect Multiply by 100. In fully adjusted models, age, sex, race, hypertension, diabetes, coronary heart disease, cancer, and tumors, BMI, HbA1c, and fasting blood glucose remained significantly associated with the odds of asthma (Table 3). In contrast, female subjects had a 39 % higher chance of developing asthma (*p* < 0.0001). Compared with Mexican Americans, non-Hispanic whites and non-Hispanic blacks were 64 % and 85 % more likely to develop asthma, respectively (*p* < 0.0001). Compared with hypertension, diabetes, coronary heart disease, and cancer tumors, the chances of developing asthma in non-hypertensive, non-diabetic, non-coronary heart disease, and non-cancer tumor patients were reduced by 32 %, 36 %, 32 %, and 27 %, respectively. Another p is <0.0001. Each unit increase in BMI (1.03), Glycated hemoglobin (1.08), and fasting glucose (1.34) was associated with a 3 %, 8 %, and 34 % increase in the incidence of developing asthma, p-value are < 0.0001, 0.0006, and 0.0013.

**Table 3:** Multivariate logistic regression model for asthma.

Variables	OR (95 % CI)	p-value
Systemic immunity-inflammation index	1.03 (1.01, 1.04)	<0.0001
Age(year)	0.99 (0.99, 1.00)	<0.0001
Gender		
Male	1.00	‒
Female	1.39 (1.24, 1.56)	<0.0001
Race/Ethnicity		
Mexican American	1.00	‒
Non-Hispanic White	1.64 (1.34, 2.01)	<0.0001
Non-Hispanic Black	1.85 (1.50, 2.28)	<0.0001
Other	1.67 (1.36, 2.04)	<0.0001
High blood pressure		
Yes	1.00	‒
No	0.68 (0.60, 0.76)	<0.0001
Diabetes		
Yes	1.00	‒
No	0.64 (0.55, 0.74)	<0.0001
Coronary heart disease		
Yes	1.00	‒
No	0.68 (0.53, 0.88)	0.0036
Cancer or malignancy		
Yes	1.00	‒
No	0.73 (0.61, 0.87)	0.0003
Alcohol drink	1.00 (0.99, 1.00)	0.5106
Energy (kcal)	1.02 (0.99, 1.05)	0.1182
Sugar (g)	1.12 (0.95, 1.31)	0.1642
Vitamin C (mg)	1.05 (0.98, 1.11)	0.1427
Magnesium (mg)	0.97 (0.89, 1.05)	0.4164
Body Mass Index (kg/m^2^)	1.03 (1.02, 1.04)	<0.0001
Glycated hemoglobin (mg/dL)	1.08 (1.03, 1.13)	0.0006
Triglyceride (mg/dL)	1.02 (0.95, 1.10)	0.5890
Total cholesterol (mg/dL)	0.94 (0.83, 1.08)	0.3794
Fasting Glucose (mg/dL)	1.34 (1.12, 1.59)	0.0013

**Fig. 2:**
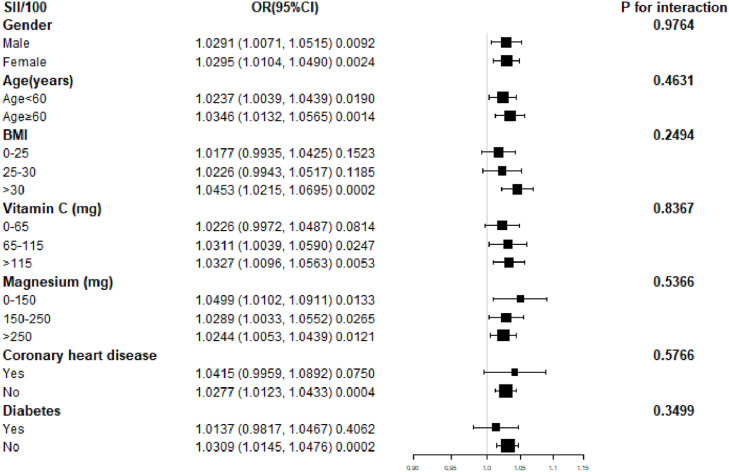
Subgroup analysis of the correlation between SII and asthma.

Fig. 2 Subgroup analysis of the association between Systemic Immune-Inflammation Index (SII) and asthma. The forest plot displays Odds Ratios (ORs) and 95 % Confidence Intervals (95 % CIs) for the association between SII (per 100-unit increase) and asthma across various subgroups. Horizontal axes represent the effect size (OR), with the vertical line indicating the null value (OR = 1.00). SII was divided by 100 to improve numerical stability and interpretability. Subgroups include gender, age (< 60 and ≥ 60-years), Body Mass Index (BMI), vitamin C intake, magnesium intake, coronary heart disease, and diabetes status. The size of each square reflects the weight of the subgroup in the analysis. Confidence intervals are shown as horizontal lines. The p-values for interaction values are based on multiplicative interaction terms in stratified multivariable logistic regression models, assessing whether associations between SII and asthma differed significantly across subgroups.

**Fig. 3:**
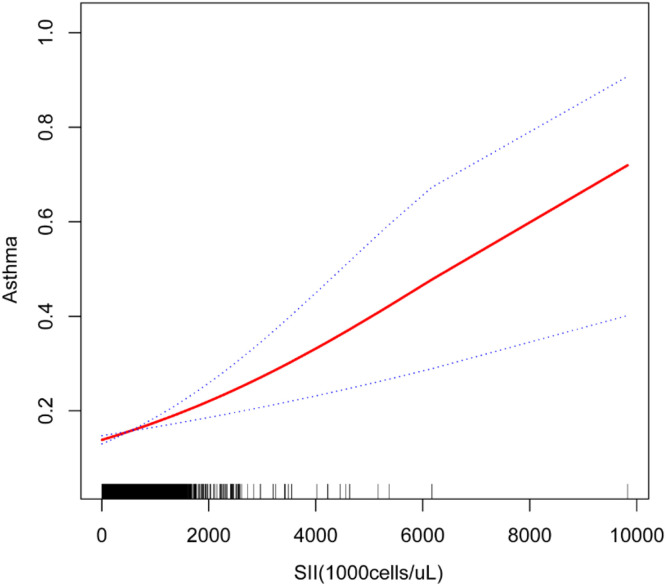
The relationship between SII and asthma. The solid red line represents a smooth curve fitting between variables. The blue band represents the 95 % Confidence Interval of the fitting.

Fig. 3 Nonlinear association between Systemic Immune-Inflammation Index (SII) and asthma prevalence. The solid red line represents the smoothed fit from a Locally weighted Scatterplot Smoothing (LOESS) regression (span = 0.7), with the blue band indicating the 95 % Confidence Interval derived from 1000 bootstrap iterations. The X-axis shows SII levels (calculated as [platelet count × neutrophil count] / lymphocyte count, expressed as ×10^3^ cells/μL). The Y-axis displays the predicted probability of asthma on a logistic scale. A segmented regression model identified a statistically significant inflection point at SII = 4.2 × 10^3^ cells/μL (95 % CI 3.8–4.6; *p* = 0.003 by Davies’ test). Below this threshold, each 1-unit increase in SII was associated with a 12 % higher asthma risk (adjusted OR = 1.12, 95 % CI 1.05–1.20); above the threshold, the association plateaued (adjusted OR = 1.01, 95 % CI: 0.98–1.04). All models were adjusted for age, sex, and smoking status. The nonlinearity test was significant (*p* < 0.01, generalized additive model).

**Fig. 4:**
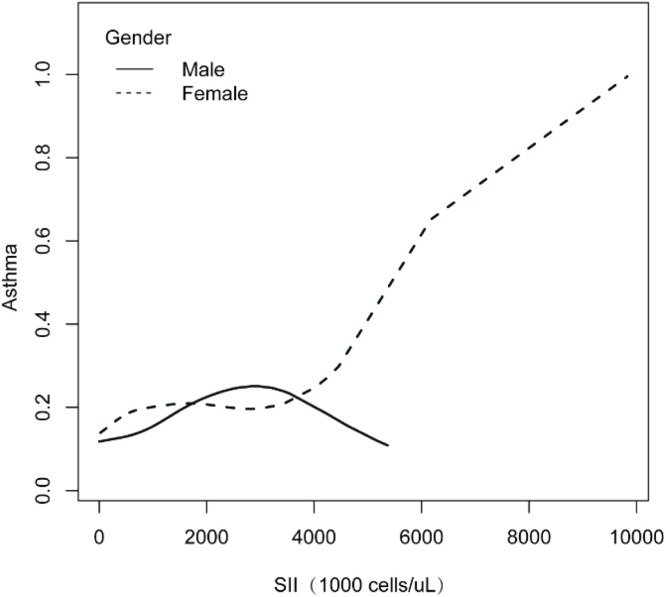
The relationship between SII and asthma is stratified by gender.

Fig. 4 illustrates the gender-stratified relationship between the Systemic Immune-Inflammation Index (SII, in 1000 cells/µL) and asthma prevalence. Non-parametric smoothing was used to fit separate curves for males (solid line) and females (dashed line). For males, segmented regression and turning point analysis indicated potential inflection points around SII values of 2000 and 4000 (1000 cells/µL), suggesting a complex, non-linear association between SII and asthma in this subgroup. The dashed line depicts the trend for females.

## Discussion

In this cross-sectional study involving 13,334 participants, the authors found that individuals with higher SII levels had a greater likelihood of having asthma. Specifically, for each 100 unit increase in SII, the odds of having asthma increased by 3 % (OR = 1.03; 95 % CI 1.01–1.04), indicating a statistically significant and positive association. In contrast, each one-year increase in age was associated with a 1 % decrease in the odds of asthma (OR = 0.99; 95 % CI 0.99–1.00). Interaction tests and subgroup analyses revealed no significant effect modification by variables such as alcohol consumption, total energy, sugar, vitamin C, magnesium intake, triglycerides, and total cholesterol (p for interaction > 0.05). Meanwhile, the diagnostic potential of SII in identifying asthma further underscores its clinical relevance.

To our knowledge, this is the first study exploring the SII-asthma relationship using NHANES data. Other studies have examined the association between inflammation and asthma in various populations with differing results and methods.^1^ Arvind Kumar et al. reported a positive correlation between hs-CRP and pediatric asthma,^16^ while Yih-Chieh S. Chen et al. suggested that higher CRP levels during pregnancy may predispose children to asthma.^17^ Terufumi Shimoda et al. found the nitric oxide fraction more effective than hs-CRP in distinguishing asthma types.^18^ Mahmoud Monadi et al. reported that asthma severity was independently associated with hs-CRP > 0.018 mg/L.^19^ Notably, most studies on CRP and asthma focus on patients under 20. Both CRP and hs-CRP have shown diagnostic value for asthma, while SAA1 has also been linked to lung diseases. Tra Cao Thi Bich et al. found that SAA1 influences neutrophilic asthma phenotypes,^20^ and Joo Rufo et al. confirmed this link in older adults.^21^ These studies support the relationship between inflammatory markers and asthma across age groups. Pediatric and adult asthma differ, with inflammation playing different roles in each. Corticosteroids are more effective in pediatric asthma due to stronger anti-inflammatory effects. Thus, age-related differences may contribute to the variation in inflammation-asthma associations.

Tuba Erdogan et al. examined the link between SII and NSAID-Exacerbated Respiratory Disease (NERD) in 105 patients,^22^ finding a 92.65 % asthma probability in those with low SII. Elizabeth Benz et al. also reported that higher SII increased asthma risk,^23^ aligning with the present results. Overall, prior research supports a connection between inflammation and asthma, both local and systemic. The present findings further confirm that SII is associated with asthma.

SII, widely used in clinical cross-sectional studies with NHANES data, has shown predictive value in diseases like hypertension,^24^ hyperlipidemia,^25^ rheumatoid arthritis,^26^ kidney stones,^27^ abdominal aortic calcification,^28^ NAFLD,^29^ and depression.^30^ CRP and procalcitonin are effective sepsis markers in neonates.^31^ NLR predicts COVID-19 prognosis,^32^ while CRP is a known acute-phase protein in pneumonia.^33^ SII has outperformed traditional markers in some contexts.^34^ In asthma research, Erdogan found that patients with an SII of 895.6 had a 30.56 % NERD risk, while those with lower SII had a 92.65 % asthma probability.^22^

Mechanistically, the present study found significantly elevated SII in asthmatic patients, likely due to increased platelet counts from systemic inflammation.^35^ Smoking-related neutrophil inflammation and oxidative stress are also linked to asthma.^36^ Erdogan's study was the first to examine SII and asthma in 105 patients, while the authors used data from 13,334 NHANES participants. Blood eosinophils and NLR are known asthma phenotype markers, and viral infections often trigger airway inflammation in children.^37^ The main strength of the present study lies in the large, nationally representative NHANES sample, which enhances the generalizability and reliability of the findings. To our knowledge, this is the first study to investigate the association between SII and asthma, highlighting the potential utility of SII as a low-cost, non-invasive inflammatory marker. However, several limitations should be acknowledged. First, the cross-sectional design precludes any inference of causality. Second, although the authors adjusted for a range of potential confounders, unmeasured variables ‒ such as genetic predisposition, environmental exposures, and lifestyle factors ‒ may still influence the observed associations. Third, medication data, including corticosteroids, beta-blockers,^38^ NSAIDs,^39^ and antihistamines,^40^ were not available in the dataset, limiting the ability to assess their potential impact on asthma outcomes. Fourth, asthma status was self-reported, which may introduce recall or reporting bias. Lastly, the ethnic diversity inherent in the NHANES cohort, while a strength in representation, may also contribute to heterogeneity in asthma phenotypes and inflammatory responses, thus affecting the generalizability of the present findings across populations.

## Conclusion

The present study found that higher SII levels are associated with an increased prevalence of asthma. Further prospective studies are needed to confirm these associations.

## Ethics statement

Reporting of the study conforms to the STROBE statement, along with references to the STROBE statement (https://strobe-statement.org/). All procedures involving human participants were approved by the Research Ethics Review Board of the National Center for Health Statistics (protocol numbers: 2018-01, 2011-17).

## Funding

This research did not receive any funding from any agency in the public, commercial, or not-for-profit sectors.

## CRediT authorship contribution statement

**Cheng Peng:** Conceptualization, Data curation, Formal analysis, Methodology, Visualization, Writing – original draft. **Dong Gao:** Data curation, Investigation, Methodology, Project administration, Supervision, Validation. **Zanchen Zhou:** Data curation, Investigation, Methodology, Project administration, Supervision, Validation. **Tiancheng Wang:** Conceptualization, Methodology, Project administration, Resources, Supervision, Validation, Writing – review & editing.

## Declaration of competing interest

The authors declare that they have no known competing financial interests or personal relationships that could have appeared to influence the work reported in this paper.

## Appendix Supplementary materials

Image, application 1
