# Supplementary material for: The hidden connection: systemic immune-inflammation index and its role in asthma
Source: Clinics (Sao Paulo). 2025 Sep 6;80:100779. doi: 10.1016/j.clinsp.2025.100779 (PMC12450745; doi:10.1016/j.clinsp.2025.100779)
Supplement: Supplementary file 1 [file mmc1.docx]

**CLINICS-D-24-01430_Supplementary Material**

**Appendix Table A1** Weighted features of SII based research population.

| **Characteristic** | **SII Quartiles** | | | | **p-value** |
| --- | --- | --- | --- | --- | --- |
|  | **Q1**  **(n = 3236)** | **Q2**  **(n = 3234)** | **Q3**  **(n = 3237)** | **Q4 (n=3236)** |  |
| Age (year) | 50.43 ± 17.20 | 49.99 ± 17.48 | 50.27 ± 17.52 | 51.44 ± 17.94 | 0.006 |
| Gender (%) |  |  |  |  | <0.001 |
| Male | 53.83 | 50.28 | 45.78 | 43.08 |  |
| Female | 46.17 | 49.72 | 54.22 | 56.92 |  |
| Race/Ethnicity (%) |  |  |  |  | <0.001 |
| Mexican American | 11.68 | 15.40 | 15.54 | 13.78 |  |
| Non-Hispanic White | 23.61 | 33.15 | 36.86 | 43.39 |  |
| Non-Hispanic Black | 37.24 | 22.39 | 18.94 | 16.66 |  |
| Other | 27.47 | 29.06 | 28.66 | 26.17 |  |
| Marital status (%) |  |  |  |  | 0.014 |
| Married | 54.73 | 57.30 | 56.19 | 53.77 |  |
| Other | 45.18 | 42.64 | 43.72 | 46.17 |  |
| Unclear | 0.09 | 0.06 | 0.09 | 0.06 |  |
| Fish eaten during the past 30-days (%) |  |  |  |  | <0.001 |
| Yes | 71.22 | 68.96 | 67.96 | 64.52 |  |
| No | 27.45 | 29.74 | 30.84 | 33.87 |  |
| Unclear | 1.33 | 1.30 | 1.20 | 1.61 |  |
| High blood pressure (%) |  |  |  |  | <0.001 |
| Yes | 36.50 | 35.41 | 36.98 | 42.99 |  |
| No | 63.44 | 64.47 | 62.77 | 56.95 |  |
| Unclear | 0.06 | 0.12 | 0.25 | 0.06 |  |
| Diabetes (%) |  |  |  |  | <0.001 |
| Yes | 13.44 | 14.78 | 15.54 | 17.99 |  |
| No | 83.44 | 82.19 | 82.30 | 79.36 |  |
| Unclear | 3.12 | 3.03 | 2.16 | 2.66 |  |
| Cancer or malignancy (%) |  |  |  |  | <0.001 |
| Yes | 8.47 | 8.87 | 9.79 | 13.32 |  |
| No | 91.47 | 91.09 | 90.18 | 86.56 |  |
| Unclear | 0.06 | 0.03 | 0.03 | 0.12 |  |
| Smoked (%) |  |  |  |  | <0.001 |
| Yes | 39.65 | 41.09 | 41.33 | 45.21 |  |
| No | 60.35 | 58.91 | 59.67 | 54.79 |  |
| Ratio of family income to poverty | 2.53 ± 1.62 | 2.56 ± 1.62 | 2.59 ± 1.63 | 2.46 ± 1.58 | 0.017 |
| Protein (gm) | 81.09 ± 42.79 | 81.91 ± 43.19 | 79.11 ± 40.63 | 76.79 ± 42.32 | <0.001 |
| Dietary fiber (gm) | 17.08 ± 11.09 | 17.14 ± 10.90 | 16.39 ± 10.56 | 15.90 ± 10.02 | <0.001 |
| Cholesterol (mg) | 319.22 ± 256.23 | 318.37 ± 257.93 | 308.75 ± 246.41 | 305.26 ± 257.86 | 0.013 |
| Vitamin C (mg) | 84.97 ± 96.07 | 80.62 ± 97.88 | 74.23 ± 81.36 | 75.81 ± 94.53 | <0.001 |
| Magnesium (mg) | 298.66 ± 149.96 | 300.38 ± 155.93 | 289.29 ± 143.74 | 285.88 ± 151.60 | <0.001 |
| Zinc (mg) | 10.55 ± 6.68 | 10.91 ± 7.98 | 10.48 ± 6.42 | 10.47 ± 10.71 | 0.016 |
| Weight (kg) | 81.18 ± 20.36 | 81.77 ± 21.12 | 83.79 ± 23.22 | 85.17 ± 24.78 | <0.001 |
| Body Mass Index (kg/m^2^) | 28.83 ± 6.46 | 29.36 ± 6.78 | 30.33 ± 7.41 | 30.94 ± 8.36 | <0.001 |
| Triglyceride (mg/dL) | 105.48 ± 103.69 | 114.73 ± 105.21 | 117.13 ± 98.28 | 109.22 ± 69.16 | <0.001 |
| Total Cholesterol (mg/dL) | 187.87 ± 41.74 | 188.88 ± 41.72 | 187.46 ± 39.79 | 185.53 ± 41.91 | 0.019 |
| Fasting Glucose (mg/dL) | 109.92 ± 36.63 | 114.48 ± 39.57 | 115.22 ± 41.11 | 114.91 ± 38.99 | <0.001 |
| Cholesterol (mg/dL) | 189.06 ± 42.01 | 190.35 ± 42.34 | 188.74 ± 40.47 | 186.80 ± 42.70 | 0.009 |
| Triglycerides (mg/dL) | 139.93 ± 127.61 | 149.63 ± 118.31 | 152.37 ± 119.92 | 143.48 ± 104.47 | <0.001 |
| Uric acid (mg/dL) | 5.46 ± 1.43 | 5.39 ± 1.42 | 5.42 ± 1.48 | 5.36 ± 1.51 | 0.005 |
| Alcohol drink | 5.06 ± 28.96 | 4.31 ± 8.31 | 4.48 ± 19.96 | 4.18 ± 4.66 | 0.935 |
| Asthma (%) |  |  |  |  | 0.002 |
| Yes | 13.81 | 14.75 | 15.60 | 17.12 |  |
| No | 86.19 | 85.25 | 84.40 | 82.88 |  |

Mean ± SD for continuous variables: the p-value was calculated by a weighted linear regression model. % for categorical variables: the p-value was calculated by a weighted Chi-Square test. Q, Quartile.
